# Use of Super Paramagnetic Iron Oxide Nanoparticles as Drug Carriers in Brain and Ear: State of the Art and Challenges

**DOI:** 10.3390/brainsci11030358

**Published:** 2021-03-11

**Authors:** Caroline Guigou, Alain Lalande, Nadine Millot, Karim Belharet, Alexis Bozorg Grayeli

**Affiliations:** 1 Department of Otolaryngology-Head and Neck Surgery, Dijon University Hospital, 21000 Dijon, France; alexis.bozorggrayeli@chu-dijon.fr; 2 ImVia Laboratory, EA 7535, Université Bourgogne Franche-Comté, 21079 Dijon, France; alain.lalande@u-bourgogne.fr; 3 Laboratoire Interdisciplinaire Carnot de Bourgogne, UMR 6303, CNRS, Université Bourgogne Franche-Comté, BP 47870, 21078 Dijon, France; nadine.millot@u-bourgogne.fr; 4 Laboratoire PRISME, JUNIA Campus Centre, 36000 Châteauroux, France; karim.belharet@junia.com

**Keywords:** iron oxide nanoparticles, drug delivery, blo-od–brain barrier, central nervous system, blood–perilymph barrier, inner ear

## Abstract

Drug delivery and distribution in the central nervous system (CNS) and the inner ear represent a challenge for the medical and scientific world, especially because of the blood–brain and the blood–perilymph barriers. Solutions are being studied to circumvent or to facilitate drug diffusion across these structures. Using superparamagnetic iron oxide nanoparticles (SPIONs), which can be coated to change their properties and ensure biocompatibility, represents a promising tool as a drug carrier. They can act as nanocarriers and can be driven with precision by magnetic forces. The aim of this study was to systematically review the use of SPIONs in the CNS and the inner ear. A systematic PubMed search between 1999 and 2019 yielded 97 studies. In this review, we describe the applications of the SPIONS, their design, their administration, their pharmacokinetic, their toxicity and the methods used for targeted delivery of drugs into the ear and the CNS.

## 1. Introduction

**Figure 1:**
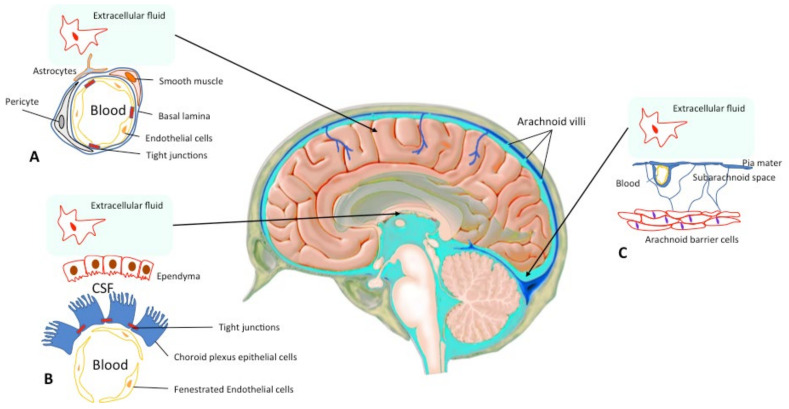
Central Nervous System Barriers. The blood–brain barrier (BBB) is present between the cerebral capillaries and the brain parenchyma (**A**). Endothelial cells with tight junctions, basal lamina, pericytes and astrocyte endfeet separate the blood compartment from the extracellular fluid. The blood–cerebrospinal fluid (CSF) barrier lies at choroid plexus in the lateral, 3rd, and 4th ventricles of the brain (**B**). The arachnoid barrier (**C**), close to the sagittal sinus, is formed by a multilayer epithelial structure with tight junctions. Arachnoid villi cross the dura, project into the sinus, and drain CSF into the blood (according to Abbott et al. 2010; Haines DE 1991 [2,3]).

Administration and diffusion of drugs in the central nervous system (CNS) is a technical and pharmacological challenge, since 3 physiological barriers control the diffusion of all substances into the CNS: the blood–brain barrier (BBB), which is the main barrier, the arachnoid barrier, which is formed by the arachnoid close to the sagittal sinus, and the blood–cerebrospinal fluid barrier, which is located in the choroid plexus (Figure 1) [1,2,3].

**Figure 2:**
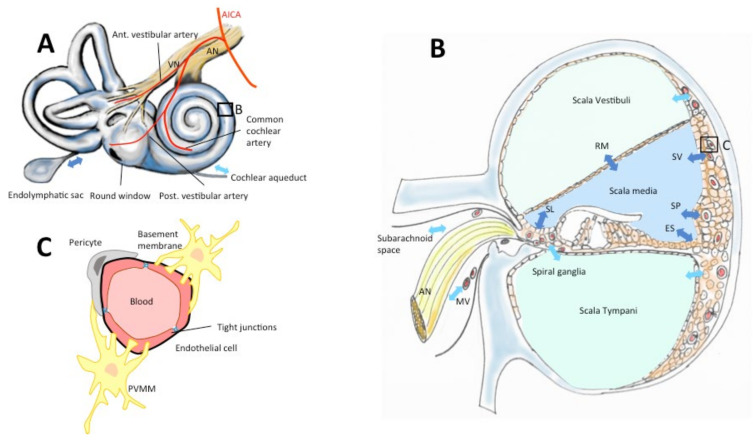
The blood–inner-ear barriers. Drugs reach the inner ear via the vascular compartment, cerebrospinal fluid or the labyrinthine windows (round, oval, **A**). The inner ear is vascularized by terminal branches of the anterior-inferior cerebellar artery (AICA, **A**). Perilymph contained in scalae vestibuli and tympani (**B**) is secreted and absorbed through the *scala vestibuli* vessels, the subarachnoid space, the modiolar vessels (MV), the capillary region in scala tympani, and the cochlear aqueduct (**A**,**B**, light blue arrows). Endolymph in the scala media is secreted and absorbed via stria vascularis (SV), Reissners’s membrane (RM), spiral limbus (SL), spiral prominence (SP), external *sulcus* (ES), endolymphatic sac (**A**,**B**, dark blue arrows). (**C**) Vessels in the inner ear epithelia are surrounded by endothelial cells with tight junctions, a double-layer basement membrane, pericytes and perivascular-resident macrophage-like melanocytes (PVMM) also called intermediate cells (according to Juhn & Ryback, 1991, Nyberg et al. 2019 [7,8]).

Generally, a high systemic drug concentration is necessary to obtain an intracerebral concentration in the therapeutic range. This exposes the patient to the risk of serious systemic complications, especially those related to chemotherapy. The same challenges exist for drug administration in the inner ear due to the presence of a blood–perilymphatic barrier (BPB, Figure 2), similar in many aspects to the CNS barriers [4,5]. The necessity to obtain a stable concentration in the therapeutic range in these compartments requires a kinetic of the delivery adapted to the elimination rate during the therapeutic period and this may require multiple daily administrations. Moreover, depending on the disease and the targets, the delivery should insure a homogeneous concentration in the organ, or on the contrary, a targeted delivery inside the compartment. Indeed, applying a drug to the vestibular organ without affecting the cochlear function to treat chronic vertigo has been one of the most important challenges in otoneurology which has not been still won [6]. Finally, the delivery method should entail minimal morbidity, especially considering the fact that the organ is already fragilized by the disease.

### 1.1. Characteristics of Blood–Brain Barrier and Blood–Perilymphatic Barrier

The BBB is the largest barrier between the brain and the endothelial system (Figure 1) [1]. The BBB mainly consists of brain microvascular endothelial cells (Bmecs), neuronal pericytes, and perivascular astrocyte foot-processes which work very closely together [1,2,7,9,10]. Bmecs and pericytes are encompassed by a basal membrane that allows them to be in an independent perivascular extracellular matrix [2]. The BBB has tight endothelial junctions (zonulae occludentes) with very high resistance (>1.8 kΩ/cm^2^) and a weak endothelial pinocytosis activity [11]. Hence, it shows low permeability, except for lipophilic molecules smaller than 400–600 Da [1,2]. Three transport mechanisms have been identified in the BBB: (1) passive diffusion following a concentration gradient and restricted to small liposoluble molecules; (2) transporter mediated and facilitated by epithelial proteins such as glucose transporters, ion channels, or exchangers; and (3) catalyzed, carrier-mediated or receptor-mediated transport by the endocytosis/transcytosis system, corresponding to an internalization of molecules by receptors through the BBB such as lectin or by electrostatic interaction [2,9]. The BBB is also active and produces enzymes to activate, inactivate or destroy many molecules (e.g., aminopeptidases, carboxypeptidases) [1]. These characteristics procure the BBB with several dynamic functions such as the regulation of ion composition in cerebrospinal fluid. Indeed, the potassium concentration is lower in the cerebrospinal fluid than in the serum (2.5 mM versus 4.5 mM) [12]. In addition, BBB protects the brain against certain macromolecules with potential proinflammatory and proapoptotic effects (i.e., albumin) [13] and neurotoxins circulating in the blood [2]. It contributes to brain metabolism by allowing water-soluble nutrients enter the brain passively [2].

Similarly, BPB plays a major role in the homeostasis of the inner ear and allows only small liposoluble or hydrosoluble molecules to pass into the perilymph (Figure 2) [4,5,7,8,14,15]. The main structures taking part in the exchanges between the blood and the inner ear are: the epithelia of the scala vestibuli, and scala tympani, the cochlear aqueduct, the subarachnoid space, the neuronal structures in the *modiolus* (for the absorption and secretion of the perilymph) and the stria vascularis, the Reissner’s membrane, the spiral *limbus*, the spiral prominence, the external *sulcus* and the endolymphatic sac for the endolymph [7,16]. The BPB is composed of anatomical elements resembling BBB, except for the vessels of the cochlear plexus and those of the stria vascularis [4,5,14]. BPB capillaries are mainly composed of an endothelium that has the particularity of being continuous with tight junctions. Cells contain intracytoplasmic microvesicles (700 A° diameter) [5] and rare micropinocytotic vesicles indicating a low transcellular vesicular transport activity. This disposition allows small molecules (<100 Daltons) to diffuse passively [8].

### 1.2. Solutions to Bypass CNS and Inner Ear Anatomical Barriers in Clinical Practice

Several procedures are employed in routine clinical practice to bypass the BBB and increase the intracerebral concentration of drugs without increasing systemic concentration [17]. Intrathecal injection can be used to deposit drugs directly into the cerebrospinal fluid. Conventional intrathecal administration requires multiple lumbar punctures and results in unsteady drug concentrations, inherent morbidity, and risk of infection. Convection-enhanced delivery diffuses a continuous intracerebral concentration of medication through microcatheters but does not avoid the risks of an in situ foreign body [18].

To circumvent some of these drawbacks, pumps have been developed. These pumps limit the risk of neural and meningeal tissue damage and reduce the risk of infection by avoiding multiple punctures. They also provide a more stable drug concentration in the CNS compartment. However, they can create a drug concentration gradient from the pump to the administration site due to the distance of diffusion, and the device could be responsible for infections in the long term [17,19]. These pumps are activated by manual pressure (Ommaya®, New York, NY, USA; Infusaid®, Norwood, MA, USA) or by an osmotic gradient (SynchroMed®, Dublin, Ireland).

Implants which gradually diffuse drugs from an intrathecal or an intraparenchymal site are also an interesting option to maintain sufficient local concentrations for several weeks, but they have the disadvantage of not being rechargeable. Gliadel® (7.7 mg Carmustine, Gliadel Wafer, Arbor Pharmaceuticals, LLC, Atlanta, GA, USA) is an implant for intracranial use, containing carmustine, a nitrosourea alkylating agent, and polifeprosan, a biodegradable copolymer used to control the release of carmustine. After resection, 1 to 8 implants can be placed in the brain tumor site, diffusing therapeutic levels of the drug for 5 days [20].

For inner ear diseases, local drug administration is routinely performed in 2 major indications: Mnière’s disease and sudden sensorineural hearing loss (SSNHL) [21,22]. The most widely employed technique for local administration for the inner ear is the transtympanic injection [23]. Under local anesthesia, the drug is injected into the middle ear cavity through a puncture in the tympanic membrane. The patient generally lies in a supine position for several minutes. The therapeutic agent in contact with the round and oval windows can bypass the BPB and enter the perilymphatic compartment, but most of the drug is evacuated by the auditory tube into the rhinopharynx. The second obstacle before reaching the inner ear is the round window membrane (RWM). This membrane is hidden in the round window niche and can be covered by a false membrane. It is ovoid with an average surface of 2.3 mm × 1.87 mm [24], and a thickness of 50–100 μm [25]. A fibrous ring attaches this membrane to the surrounding promontory bone [25]. The RWM separates the perilymph of the scala tympani from the air in the middle ear, and its size, form and accessibility are highly variable in humans [26]. It is semi-permeable and made of 3 layers: internal and external epithelial layers separated by connective tissue [25,27]. The external epithelial layer contains tight junctions and a basal lamina [25,27]. In this layer, the epithelial cells are equipped for active transepithelial transport (microvilli, mitochondria, rough endoplasmic reticulum). However, the internal epithelium lacks continuity. Its cells are connected with loose junctions and pinocytotic vesicles are present, indicating transepithelial transport [27]. These characteristics allow only lipophilic molecules or those with a low molecular charge to cross the RWM [27]. Hence, the perilymphatic concentration of dexamethasone-21-dihydrogen-phosphate, the most frequently employed drug, varies from less than 0.01 to 6% of the solution applied to the RWM [28]. Because of the interindividual variability of this gateway, drug diffusion to the inner ear is difficult to evaluate [29]. Moreover, the perilymphatic flow inside the inner ear is negligible, and drugs penetrating through the RWM accumulate in the basal turn of the cochlea, creating a base-apex concentration gradient (base/apex concentration ratio of 17,000 in rats) [28].

To circumvent the limitations of an intratympanic injection, several solutions have been attempted at a clinical stage. In 23 patients with an idiopathic SSNHL resistant to systemic steroid therapy, a silicon microcatheter (Round Window mCathTM, Durect Corp., Cupertino, CA, USA) was placed through the RWM a few mm into the basal turn and methylprednisolone-hydrogen succinate sodium salt or dexamethasone–dihydrogenphosphate were injected continuously by an external electronic pump (Panomat C5, Disetronic Medical Systems, Burgdorf, Switzerland) for 4 weeks [30]. Complications such as catheter dislocation, granulation tissue in the middle ear, ear canal skin defects, and small defects of the tympanic membrane were frequent (78%). This intracochlear mode of administration was highly effective since no further hearing deterioration was noted, and the improvement of the audiometric pure-tone-threshold ranged from 16 to 87 dB (pure-tone-average 103 dB before treatment versus 87 dB after).

Another approach aimed at increasing the bioavailability of the drug at the RWM without perforating it. Dexamethasone was mixed with poloxamer 304 (Otovidex, Otonomy, San Diego, CA, USA) and injected into the middle ear [31]. Poloxamer 304, which is liquid at ambient temperature, solidifies at 37° in the middle ear cleft and progressively liberates the drug in contact with the RWM for several days. The poloxamer 304 was injected in a single transtympanic injection. The use of this drug has been attempted in 44 patients with unilateral Ménière’s disease with few minor complications (tympanic membrane perforation, pain), with no impact of hearing function and fair efficacy on vertigo frequency (73% reduction in vertigo frequency with 12 mg of poloxamer 304-dexamethosone mix, 56% with 3 mg of the mix, versus 42% with placebo) with a 3-month follow-up [31].

In cochlear implants, dexamethasone was added to the silicone surrounding the electrode carrier. The aim of this device was to obtain a passive diffusion of the drug into the scala tympani and to limit inflammation due to surgery [32]. In vitro assessment of these electrode arrays showed that 1–5 μg of dexamethasone were released during the first 24 h. The diffusion followed 2 phases: a rapid burst during the first 50 days and a steady release period from day 50 to 700. This type of array has not been evaluated in a clinical trial. Deep-seated catheters into the cochlea could be another method of administration close to the target structures with no intracochlear gradient [32]. They could be combined to a cochlear implant and imbedded in the electrode array. As a proof of concept, a flexible microcannula (0.5–0.8 mm diameter) was introduced 15 to 20 mm in the scala tympani to allow the administration of 10 µL of a contrast agent [33]. The limitations of this technique are the possible physical modifications of electrode characteristics (larger diameter) and consequent trauma, potential increase in intracochlear hydrostatic pressure, and the necessity to resupply the pump.

### 1.3. Why Use Superparamagnetic Iron Oxide Nanoparticles (SPIONs) to Bypass Biological Barriers?

SPIONs are a promising option since their superparamagnetic properties allow them to be guided by magnetic forces to deliver the drug to the target with precision. In addition, they can be designed to include theranostic properties. Their surface-to-volume ratio can be significantly increased by the use of shells, and the number of ligands (targeting antibody or peptide), surface charge, hydrophobicity and biocompatibility can also be modified [34,35].

The main application of SPIONs is in diagnostic imaging [36,37]. SPIONs are used as contrast agents in MRI. Several molecules are approved by the Food and Drug Administration (FDA) for routine clinical use: Dextran-coated Iron-oxide (Ferumoxtran), Carboxydextran-coated Iron-oxide (Ferucabotran), and Polyglucose sorbitol carboxymethylether-coated iron oxide (Ferumoxytol) in lymph node, hepatocellular carcinoma cell, monocellular phagocyte system labeling and imaging [38].

More importantly, SPIONs can represent an efficient means of drug delivery as we will develop in this review [39,40]. SPIONs are particularly interesting for drug delivery in CNS and inner ear since both structures are guarded by a blood–organ barrier, contain deep-seated, functionally sensitive, and fragile structures [41], and can benefit from targeted deliveries using an external magnetic field (EMF) [42,43,44,45]. Modifying their surface charge, combining them to hydrophile molecules (e.g., Polyethylene Glycol, PEG; Polyethylenimine, PEI; Polylactic-co-glycolic acid, PGLA) [40,46] or coupling them to antibodies (transferrin receptor antibodies or lactoferrin) [47,48] can further facilitate their diffusion through BBB [49,50].

## 2. Materials and Methods

**Figure 3:**
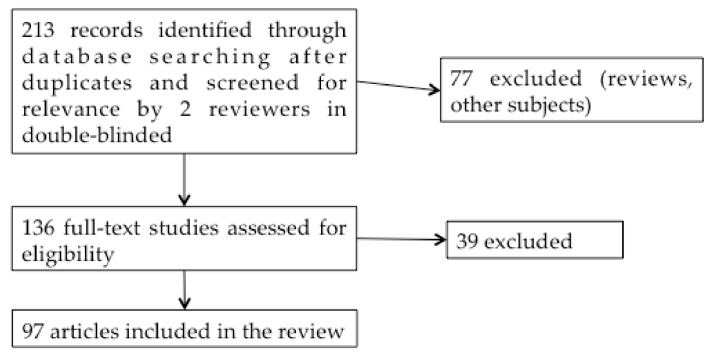
Systematic review flow diagram.

In this double-blind systematic review study, we initially included 213 studies published between 1999 and 2019 in English on the PubMed website, using the keywords: “Superparamagnetic iron oxide nanoparticles”, “Ear”, “Central Nervous System” or “Cerebral” (Figure 3).

Articles studying only the use of SPIONs as a magnetic resonance imaging (MRI) contrast agent were not included in the study. From the titles and abstracts, 136 studies were selected by the 2 reviewers. Reviews were excluded. After reading these 136 articles, 97 reports on the application of SPIONs to drug administration were finally included in the review. Additional articles concerning SPIONs shell characteristics, BBB and BPB were also added to this work to clarify the purpose.

## 3. SPIONs Characteristics

**Figure 4:**
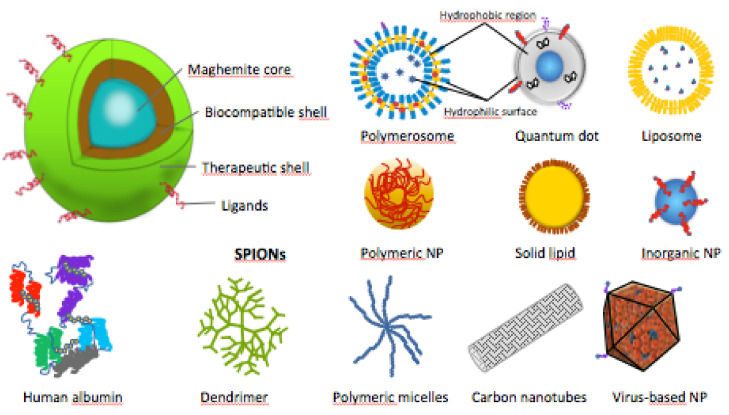
Schematic structures of SPIONs and other types of therapeutic nanoparticles (NPs).

The SPIONs’ cores are composed of magnetite (Fe_3_O_4_) or maghemite (γ-Fe_2_O_3_), and are encapsulated in organic or inorganic shells, to increase their biocompatibility and enhance their in vivo applications (Figure 4) [35]. In some studies, SPIONs were used naked or their shells were not specified [44,51,52,53,54,55].

The composition of the shells is important because it influences the interaction between SPIONs and the medium in which they are placed [34,35]. These interactions may lead to the formation of SPION clusters, cellular and tissular adhesion, barrier crossing, and indirectly their magnetic susceptibility.

### 3.1. Organic Shells

Polymer coatings are mainly used to limit the agglomeration of SPIONs due to the magnetic and Van der Waals forces (Table 1).

Cationic polymers are privileged since they are biocompatible and have a good absorption capacity. They are interesting for drug delivery in brain, especially for antitumor drugs, because they lead to negative charges at the surface of nanoparticles (NPs) to promote their intratumoral absorption or facilitate the passage of BBB via mediated adsorptive transcytosis [105]. Most cationic polymers, such as chitosan or polyethyleneimine (PEI), have amino groups in their structures that can be protonated at acidic pH. They can also be conjugated by various ligands with amine or carboxyl groups for targeted administrations.

PEI has an electrostatic repulsion effect which decreases the hydrodynamic size of SPIONs modified with this molecule and their dispersity index [40,56,57,58,59,73,74,106]. The efficacy of fluorescent magnetic PEI-poly(lactic-co-glycolic-acid) (PLGA) NPs loaded with paclitaxel (PEI-PLGA-PTX-SPIONs) was studied by assessing apoptosis of human glioblastoma (GBM) cell line U251 in vitro with different concentrations for 12 h. It has been showed that autophagy and cell apoptosis were more important with the higher concentration (400 μg/mL) and with PEI-PLGA-PTX-MNPs (70% of rate apoptosis) versus PLGA-PTX-MNPs (50%) or PEI-PLGA-MNPs (0%) [74]. The results indicated that PEI increased the drug effect by enhancing the intracellular drug bioavailability.

Polyethylene glycol (PEG) provides both hydrosoluble and liposoluble characteristics to the particles, maintains electroneutrality, and avoids recognition by the reticulo-endothelial system to increase blood circulation time [107]. These properties have been exploited in multiple studies [18,40,47,50,56,57,58,59,60,61,62,63,64,65,66,67,68,69,70,71,72]. The efficiency of doxorubicin (DOX) and indocyanine green loaded on 1,2- distearoyl-sn-glycero-3-phosphoethanolamine-N-PEG-SPIONS (SPION@DSPE-PEG/DOX/ICG) was compared to free DOX and SPION@DSPE-PEG NPs on glioma tumors in rats (n = 38) after the confirmation of their in vitro biocompatibility [66]. The NPs were administered with a tail vein injection with any external magnetic field. At 8 h, the signal on fluorescence imaging and MRI was increased in the tumor site with SPION@DSPE-PEG/DOX/ICG in comparison to the free indocyanine green. This was in favor of a better diffusion through the BBB. From days 7 to 21, the tumor size decreased significantly with SPION@DSPE-PEG/DOX/ICG compared to other groups. These results confirmed the efficiency of SPION@DSPE-PEG/DOX/ICG on glioma tumor on rats.

Like PEG, PLGA is also used as a shell for SPIONs and can carry at the same time both hydrophobic and hydrophilic molecules. It is biodegradable and FDA-approved [43,46,63,74,75,76,77,78,108]. Small variations in particle sizes of PLGA lead to changes in the efficacy of NPs in delivering drugs [75]. This combination with PEG and transferrin enhances the transport through BBB and increases intracerebral bioavailability [63]. Observations in nude mice with GBM injected with SPIONs delivering temozolomide show that the addition of antibodies against nestin receptors on the same coating allows the NPs to increase their intracellular penetration and drug efficacy [63].

Dextran and carboxy-dextran were also considered as constituents of the SPIONs’ shell [80,88,89,90,91,92,93,94,95,96,97,98,99,100,101,102,103,104]. Dextran is a very high-molecular-weight branched polymer of dextrose (glucose) with colloid properties. It can be used with other coatings (including surfactant and oleic acid) to increase blood circulation time and loaded in monocyte-derived macrophages to increase biocompatibility and transport through BBB without toxic effects on brain cells in a mouse model [102]. Cross-linked aminated dextran (CLIO-NH_2_) was also assessed as a potential SPION coating. The SPIONs with such coating elicited no side effect in zebrafish below 200 mg/kg during 24 h, suggesting good biocompatibility [88].

Other polymers derived from glucose such as D-mannose [85] rhamnose (mannose deoxyhexose) [109], arabic gum (sap exudate) [110] and hydrophilic polysaccharide matrix of starch [111] also showed promising results in vitro (Table 2). SPIONs coated with a rhamnose derivative on 2 GBM cell lines (T98G and U251) and other tumor cells such as human urinary bladder carcinoma cell line (ECV304) and fibroblast cell line (BALB/3T3) showed an increased cellular uptake and dose-dependent cell death after incubation for 24 h (e.g., for T98G cells: 12% of death at 1 μg.Fe^−1^.mL versus 35% at 100 μg.Fe^−1^.mL) [109].

Chitosan is a hemocompatible, biocompatible, and hydrophilic polyoside composed of D-glucosamine and N-acetyl-D-glucosamine (a natural cationic linear polymer) and offers active sites for combination with PEG and PEI [36,40,59,68,87]. SPIONs coated with chitosan-PEG copolymer and bearing O_6_-benzylguanine and a targeting peptide, chlorotoxin (NPCP-BG-CTX) were used to potentiate the effect of temozolomide by decreasing the upregulation of the DNA-repair protein O6-methylguanine-DNA methyltransferase (MGMT) [68]. In human GBM cell lines, they decreased the MGMT activity and increased the efficacy of temozolomide. In mice with GBM, these SPIONs co-administered with temozolomide led to a 3-fold increase of the survival with the convection-enhanced delivery (the administration was performed with a stereotaxic frame at the same localization for the tumor brain implantation). Follow-up MRIs showed an enhanced intracerebral diffusion. This type of drug delivery uses fluid convection to create a pressure gradient during interstitial infusion, a better volume distribution, and mobilize NPCP-BG-CTX [68].

Other examples of organic shells including polyvinyl alcohol (PVA) which prevents particles coagulation [80,81,82], aniline-co-N-(1-one-butyric acid) aniline (SPAnH) [83], Poly(γ-GA-co-DSGA) [76], polybutulcyanoacrylate (PBCA) [84], and poly-(dimethylamine-co-epichlorhydrin-co-ethylendiamine) (PEA) [86] showed promising results but the evaluation is still at the in vitro stage.

Polymers derived from amino acids were considered as SPIONs coating to increase their biocompatibility: gluthatione [58,62], glycine [82], glutamic acid [82], collagen [82], human serum albumin [18,82], aminosilane [112], and spermine [90] (Table 2). The ability to cross a model of BBB in vitro of 5 SPIONs with different coatings in this category: 1—glycine, glutamic acid, bovine serum albumin (GGB), 2—glycine, glutamic acid, collagen (GGC), 3—glycine, glutamic acid, polyvinyl alcohol (GGP), 4—bovine serum albumin, polyethylene glycol (BPC), 5—collagen, polyvinyl alcohol, bovine serum albumin (CPB) showed that CPB-SPIONs, coated with collagen, have the highest in vitro cellular uptake and the best diffusion levels through BBB (2.7 g/mL of CPB were found after 2 h in the receiving well by a Ferrozine assay kit versus 1.9 of BPC and GGP, versus 1.5 of GGB, and versus 1.7 of GGC) [82].

Polymers derived from lipids (oleic acid [76,81,112], micelles [48,62], DSPE [50,113], phospholipid [66], DMPC [69], and lipophilic fluorescence dye [111]) show good biocompatibility and capacity to cross biological barriers (Table 2). SPIONs coated by a lipophilic fluorescence dye were studied in vitro to evaluate their capacity to cross brain capillary endothelial cells (BCECs) with or without an external magnet with a field strength of 0.39 tesla [111]. At 140 μg/mL, the average concentration of SPIONs passing through the BCECs with a magnet was around 60 μg/mL versus 0 μg/mL without a magnet. No sign of BCECs’ toxicity was observed [111].

Monomeric citrate coating on SPIONs facilitates intracellular passage and NP accumulation in microglia and induces changes to the cellular morphology in vitro [114,115] (Table 3). 

Although the increased intracellular penetration is interesting to reach some targets, intracellular accumulation of SPIONs may be toxic and its long-term effects should be monitored. Surfactants limiting the aggregation of SPIONs and increasing their stability could also be applied on SPION surface [46,49,57,64,65,75,77,84,102]. A surfactant such as Twen 80 (or polysorbate 80, water-soluble non-ionic emulsifier) confers amphiphilic properties to SPIONs and increases their BBB crossing [46,49,57,64,77,84]. A study assessed the effect of 4 surfactants (Twen 80, Brij-35, Pluronic F68, Vitamin E- α-tocopheryl-polyetheleneglycol-succinate, TPGS) on BBB crossing [77]. The contribution of the different types allowed a crossing of the BBB with visualization of NPs on the MRI and a preserved toxicity of DOX on glial cells (glioma cell lines U87 and 9L). In all cases, the NPs structure was stable at 3 months [77]. These surfactants need further investigation in animal models.

Curcumin (diferuloyl methane) is a liposoluble molecule with anti-oxidant, anti-apoptotic, anti-inflammatory, and anti-proliferative properties [48,116,117,121]. The neuroprotective efficacy of SPIONs coated by curcumin was studied in the cerebellum of schizophrenic rats [116]. Curcumin-SPIONs reduced reactive oxygen species formation, increased succinate dehydrogenase activity, and mitochondrial adenosine triphosphate level in comparison to naked SPIONs. These observations were in favor of a neuroprotective effect of curcumin-SPIONs. 

Liposomes can be combined to SPIONs by covering one or several NPs [47,64,71,101,119,120]. A liposome is an artificial vesicle formed by concentric lipid bilayers, trapping aqueous compartments between them. It is obtained from a wide variety of amphiphilic lipids, most often phospholipids. Drugs attached to NPs can be incorporated into liposomes, increasing the drug capacity. Liposomes can also protect SPIONs from degradation enzymes in the blood and increase the bioavailability in circulation. It is possible to couple a protein (e.g., receptor-specific ligand) to the liposomal bilayers for drug delivery or achieving specific cellular recognition [122]. SPIONs with anti-CD20 (Rituximab, RTX) coated with liposome (Lip), PEG and Twen 80 (Lip/PEG/Tween80/SPIONs-PVA/RTX) were used and tested against primary CNS lymphoma in vitro on B cell lymphoma cells [64]. The ratio of cellular uptake was higher for Lip/PEG/Tween80/SPIONs-PVA/RTX than in Lip/PEG/Tween80/SPIONs-PVA in Z138C and in Granta cells line. At 7 h after incubation, the cell viability of the Grant cells line was lower with Lip/PEG/Tween80/SPIONs-PVA/RTX (60%) than in the control group and in cells with free RTX (100%, *p* < 0.05). These promising observations indicated an improvement of intracellular RTX delivery and enhanced action.

The function of exosomes has been diverted to transport drugs [117]. Their vesicles derive from late endosomes and allow extra-cellular transport of cellular components.

### 3.2. Inorganic Shells

Silica was used in one study [123] as hydroxyapatite [124]. It is an inert shell but allowing multiple bonds [125]. Gold has been used [103,126] for its versatility in surface modification without damaging the BBB and for the protection of SPIONs against oxidation. Pure carbon is also an interesting inorganic shell due to its low density, its ability to increase magnetic flow and its solidity [125,127,128]. Certain inorganic shells such as graphene were also evaluated for their biological effects at the in vitro stage [67,78]. Graphene, or graphene oxide, is a two-dimensional, flat structure with a thickness of an atom, a large surface, and excellent electrical, thermal, and mechanical properties [129]. SPIONs coated by graphene oxide and lactoferrin and loaded with doxorubicin hydrochloride (Lf@GO@Fe_3_O_4_@DOX) were engineered. Lactoferrin was used to increase the across of BBB in further in vivo studies. In vitro application of 10 μg/mL DOX with or without SPIONs on C6 glioma cells for 24 h showed encouraging results with an improvement of drug efficacy when loaded to SPIONs: cell viability was 20% with free DOX, versus 65% with GO@Fe_3_O_4_@DOX or 60% with Lf@GO@Fe_3_O_4_@DOX.

Fluorescence was incorporated in the shell composition of some SPIONs to control their intracellular movements by magnetic field and to monitor them at the same time with a fluorescence microscope [48,74,111]. These characteristics provide effective ways of identifying the specific functions of bioactive molecules in specific areas of living cells without disturbing other parts of the cell.

## 4. SPIONs Transport

### 4.1. Central Nervous System

The intracerebral drugs delivery by SPIONs was most often conducted through the BBB after systemic administration. A passive diffusion to the CNS was made possible by their liposoluble properties [65,130].

Antibodies against transferrin or lactoferrin receptors or other molecules to promote internalization were placed on the SPIONs to cross the epithelial cells by endocyte/transcytosis system [47,48,50,63,91,113]. Vitamin B6 on SPIONs was used to reach the transferrin receptor on the apex of endothelial cells [113]. Transferrin allows the endocytosis of NPs and the intracellular inhibition of glycoprotein P, which is responsible for the extrusion of foreign bodies [131]. The impact of hyperthermia associated with chemotherapy (temozolomide) on the growth of GBM cells in vitro was also studied [50]. Temozolomide was loaded with SPIONs in lipid NPs (LMNVs) with surface antibodies against transferrin receptors in order to cross the BBB and to be internalized into GBM cells. Hyperthermia was created, and lipophilic temperature-sensitive fluorescent dye was used to monitor the intraparticle temperature in response to alternative magnetic field (AMF) exposure. At 72 h, the passage of BBB was increased for NPs charged with antibodies and subjected to AMF. A synergistic effect of the 2 phenomena may weaken the BBB.

In order to increase the permeability of BBB, NPs were covered with Twen 80 (polysorbate 80, a surfactant with high solubility) [46,49,57,77]. The usefulness of Twen 80 was demonstrated in a study comparing the intracerebral concentration of PEG-PEI-SPIONS and Twen80-SPIONS in rats with the application of EMF [57]. After IV administration of the NPs, the intracerebral concentration of Twen80-SPIONs was higher than that of PEG-PEI-SPIONs, even with the use of EMF. Four types of surfactant (Twen 80, Brij-35, pluronic F68 or Vitamin E-PGS) were combined to PLGA-coated SPIONs and loaded with doxorubicin [77]. Suspensions remained stable for 3 months; vehicles without the drug showed no toxicity on glial cells, the NPs delivered doxorubicin to gliosarcoma cells (9L/lacZ gliosarcoma rat cell line) and GBM cells (human GBM U-87 MG) and increased apoptosis with SPIONs with any of the 4 surfactants [77].

Lipopolysaccharides (LPS) were also used to weaken the BBB by creating inflammation and allowing NPs to cross it [132]. In mice, 24 h after LPS administration (3 mg/kg IV), the amount of thioflavin S (fluorescent amyloid ß-binding small molecule) and 30 nm SPIONs increased in the brain, suggesting an enhanced permeation effect of the LPS. However, these observations were only found in adult mice and not in young animals, suggesting the effect of age on this permeation.

The use of an EMF made it possible to draw SPIONS through the BBB [42,43,47,49,57,60,61,73,76,78,81,83,94,113,119,120,128,133,134]. EMF (with a field strength of 0.3 tesla) were used to increase mobilization capability on SPIONs associated with the solubility of the Twen 80 to cross the BBB and guide them in the intracerebral compartment [57].

Hyperthermia was employed to increase the size of BBB pores with alternating magnetic current [50,101,117], and ultrasound (US) was also employed to break barriers at microscopic level in the BBB [52,94,135,136,137]. Ultrasound can change the permeability of cellular membranes, and by extension the permeability of the entire BBB. This phenomenon is called sonoporation. Ultrasmall SPIONs (USPIONs) surrounded by microbubbles (composed of poly(n-butyl-cyanoacrylate) and FITC-Dextran) were injected intravenously into mice and a sonoporation was conducted [137]. The microbubbles were used to amplify the sonoporation by reducing the necessary acoustic force and at the same time allowing the delivery of the drug. Results indicated an enhanced diffusion of the USPIONs to the CNS compartment when combined to microbubbles and ultrasound, and an increased permeability of BBB to large molecules (FITC-Dextran, 70 kDa).

SPIONs coated with the drug were administered directly in the targeted brain site [18,51,54,58,69,97,103,133,138,139] or diffused through the olfactory bulb to short-circuit the BBB (with or without EMF) [49,112]. After endonasal injection in mice, the efficacy of these cells in free form and carried by rhodamine B-covered SPIONs were compared in order to test the effectiveness of mesenchymal stem cells on olfactory bulb damage. Cells were guided by an EMF [112]. When the EMF was applied and the mesenchymal stem cells were transported by rhodamine B-covered SPIONs, the cell concentration in the damaged olfactory bulb was higher than it was following free injection or without the application of EMF. This approach is a promising means of delivering drugs to the brain because it is able to circumvent the constraints of the BBB without noticeable side effects.

### 4.2. Inner Ear

In order to reach the inner ear, SPIONs could be driven by an EMF though the round window [45,79], using a cochleostomy [44] or deposited in the middle ear for passive diffusion through the round window [111]. In vitro, an EMF was used to increase the migration of SPIONs in cochlear cells [62,96]. After verifying the absence of toxicity of NPs on cells derived from the inner ear (EC5V), the NPs were injected through a cochleostomy into the inner ear of guinea pigs [44]. At one week, no decrease in hearing was noticed as judged by the auditory brainstem response (ABR). This indicated the short-term good tolerance of SPIONs by the inner ear. The targeting of the inner ear with SPIONs after IV or intrathecal injections has not been reported to our knowledge.

## 5. Indications

### 5.1. Central Nervous System

SPIONs have been mainly used to carry chemotherapy agents aimed at GBM [18,36,43,48,50,51,55,59,61,63,65,66,67,68,70,72,74,76,77,78,82,83,90,92,94,95,98,99,100,101,109,110,117,119,120,125,133,136,138,140,141,142], but also for cerebral lymphomas [64] and pediatric CNS tumors [40]. DOX and temozolomide often serving as the first-line chemotherapy are the 2 main candidates combined to SPIONs. The use of a surfactant is thought to increase the biocompatibility of the NPs and in combination with PLC, PEG and folic acid increase the intracellular concentration of DOX (DOX-PCL-SPION-sPEG-FA) [65]. Interleukin-1 receptor antagonist with known anti-edematous properties coupled to SPIONs covered with dextran (SPIONS-Il-1RA) has been studied in vitro and in vivo (rats with GBM) [99]. SPIONS-Il-1RA showed no toxicity in vitro on C6 glioma cell lines and lymphocytes, had an anti-edematous action in the animal brain, and increased survival. They also produced a signal inversion of the tumor in T2-weighted MRI images indicating the presence of SPIONs in the site after IV injection and their capacity to cross BBB.

The chemotherapy effect delivered by SPIONs could be potentiated by proton therapy [92], external ionization [98] or radiation therapy [40]. In vitro, SPIONs coated with dextran, folates and paclitaxel (FA-PTX-D-SPIONS) combined with protontherapy appeared to increase the delivery of drugs (thanks to folates), to potentiate radiosensitization (by the presence of SPIONs) and to develop the action of paclitaxel (by protontherapy) on C6 rat GBM cells after the low toxicity of FA-PTX-D-SPIONS was confirmed at a concentration less than 200 ng/mL in normal cells [92].

The use of SPIONs in the management of epilepsy is another interesting application under assessment at the animal model stage [49,60,72]. In Swiss albino mice, SPIONs and nanostructured lipid carriers incorporated to a thermosensitive mucoadhesive gel and placed close to the olfactory bulb can deliver clonazepam to the target site effectively when driven by an EMF [49]. In rats with chemically-induced temporal lobe epilepsy, SPIONs carrying alpha-methyl-A-tryptophan, a surrogate marker for this disease, and inteleukin-1 beta monoclonal antibody as the therapeutic agent were administered intravenously. Histology, imaging, and molecular biology findings indicated a targeted effective drug delivery [72].

SPIONs were also applied for drug delivery in CNS degenerative diseases [54,84,89,94,113,132,139]. In rats, the ability of dextran-covered SPIONs to increase the bioavailability of quercetin in the brain has been demonstrated [89]. Quercetin, a flavonoid with potential protective effect against neurodegenerative diseases, was administered orally in a free form or attached to SPIONs. Whatever the initial concentrations (50 or 100 mg/kg), the concentration of quercetin was higher with SPIONs, suggesting better passage through the BBB.

The treatment of aneurysms and strokes also has a place in research using SPIONs [42,56]. SPIONs can be used to guide endothelial progenitor cells with an EMF and their location can then be identified in MRI imaging [42]. In vitro studies confirmed the angiogenic potential of SPIONs-endothelial progenitor cells by highlighting the creation of neovascularization and the secretion of growth factors (VEGF and FGF) by outgrowth endothelial cells in mice and humans. The SPIONs were loaded into the endothelial progenitor cells through the endosomes/lysosomes system. In mice, the nanostructure was guided in vivo by an EMF and detectable in MRI, which confirms their potential for use in cerebrovascular conditions. SPIONs could also be used for nerve regeneration, especially in combination with brain-derived neurotrophic factor [143].

### 5.2. Inner Ear

Research on the use of SPIONs for inner ear diseases is less extensive than for CNS pathology. Dexamethasone has potential indications in sudden sensorineural hearing loss, Ménière’s disease and other inflammatory conditions of the inner ear such as autoimmune diseases [21,22]. SPIONs combined to dexamethasone administered into the perilymphatic space through the round window show no toxicity in rats and guinea pigs as judged by hearing levels and histological observations [79,87]. Dexamethasone coupled to 500 nm SPIONs could diffuse to the inner ear through an intact RWM [79]. This diffusion was facilitated by a magnet (0.26 mtesla) placed on the contralateral ear [79].

The efficacy of SPIONs to reduce the ototoxicity of cisplatin was investigated in inner ear organotypic cultures of mice [62]. This approach was based on scavenging cisplatin by the NPs and protecting the cells against DNA damages and oxidative stress generated by this drug and on the ability of glutathione transported by the NPs to provide resistance to the Corti cells against cisplatin via its antioxidant properties. SPIONs were encapsulated with polymeric micelles (a glutathione diethyl ester-conjugated amphiphilic diblock copolymer) sequestering cisplatin and were guided by an EMF into the Corti cell cultures. The results showed no toxicity to the cochlear hair cells when using micelles up to 415 μM of iron concentration. The observations confirmed that the conjugated micelles are capable of sequestering cisplatin and can protect cells from its cytotoxic effects (partial protection of cochlear cell growth and decreased cell apoptosis).

## 6. Pharmacokinetic and Toxicity

SPIONs must be able to meet the requirements to deliver drugs while remaining biocompatible. These characteristics depend on their size, shell, charge, structure, concentration, biodistribution, solubility, immunogenicity, surface-to-volume ratio allowing interaction with different body components, ability to resist biodegradation, and finally ability to cross natural barriers [144,145]. In this review, several articles focused on the pharmacokinetics of NPs, which is crucial for the assessment of their bioavailability, toxicity, and adverse effects [43,66,76,83,89,90,98,113,117,118].

For in vivo applications, the optimal size of SPIONs is between 10 and 100 nm [144,145]. After subcutaneous or intratumoral administration, the NPs enter the lymphatic capillary system, except for the hydrophilic particles which penetrate through the blood capillary walls and to the blood circulation [145]. The route of choice in most studies targeting CNS is the IV injection [42,43,48,57,76,78,90]. For the inner ear, intratympanic injection is preferred since it has the advantage of delivering a large SPION quantity by a minimally invasive route [44,45,79].

After IV injection, the reticuloendothelial system (RES) eliminates the SPIONs from the circulation in 2 steps. Initially, opsonization (adsorption on plasma proteins) leads SPIONs to interact with specialized membrane receptors on monocytes and macrophages, and promotes their recognition by these cells [146,147]. The second step is the endocytosis/phagocytosis of SPIONs by the RES cells removing them from blood and increasing their concentration in organs with high phagocytic activity: 80–90% in the liver, 5–8% in the spleen and 1–2% in the bone marrow [146]. The SPION’s size influences the elimination route: those below 10 nm are eliminated from the circulation by renal clearance, but larger particles (>200 nm) accumulate in the spleen [144,145]. A study using a stem-cell model has shown that a tissue can purge itself of NPs with endosomes in one month without perturbing cellular iron homeostasis [148]. In drug delivery applications, the shell composition is important. Circulation time increases with small particles covered with a neutral and hydrophilic shell [147]. Using liposomes as shell has the disadvantage of being quickly phagocytized by RES. To counteract this process, the use of PEG with liposome could be a solution to reduce RES uptake (particularly Kupffer cells) by creating a hydrophilic membrane [146].

Specific ligands modify the pharmacokinetics significantly. Heat-shock protein (HSP) 70 is present on the membrane of many malignant cells such as GBM. Membrane HSP 70 is internalized in these cells providing an entry root for vehicles linked to this marker [98]. The biodistribution of SPIONs coated by dextran and HSP 70-specific antibody after a single IV injection was evaluated in rats by histology and MRI 24h after injection. HSP 70-specific antibody increased the SPION uptake by the glioma 7-fold compared to uncoated SPIONs. Brain radiotherapy further increased the uptake [98]. Application of EMF can also alter the pharmacokinetics of these drug vehicles. To assess this effect, SPIONs with paclitaxel encapsulated by adipose-derived stem cells (ADSCs-SPIONS-PTX) were administered intravenously in mice with GBM [76]. A high-frequency magnetic field was applied to drive SPIONs into the lesion and generate heat inside the tumor. The efficiency of thermo/chemotherapy combination was assessed. SPIONs were labeled with a near-infrared fluorophore for in vivo fluorescence detection and organs (heart, liver, spleen, kidney, tumor) were collected for ex vivo optical imaging 72 h post injection. The in vivo fluorescence detection revealed ADSCs-SPIONS-PTX accumulation only in the tumor. At 72 h, the ex vivo optical imaging showed 45% of fluorescence intensity in brain tumor, 35% in liver, 17% in lung and traces in kidney, heart and spleen. Moreover, SPIONs were not the cause of systemic or organ side effects in these different studies but the follow-up was very short.

Currently, development and study of NPs–protein corona complex could increase biodistribution, biosafety and blood circulation time [149,150]. Protein corona is composed by different protein layers and able to change NPs properties, which is detected by cells (especially those of the RES). The first layer is fixed but the second could be removed. Protein corona is a dynamic and evolutive process which is able to adsorb proteins from biological fluids and form bio-nano interface via a protein membrane. Protein corona could have different physico-chemical characteristics depending on SPIONs (size, charge, surface) and environment properties. During adsorption of proteins, conformational changes are made: proteins have structural rearrangements resulting in NPs surface changes. This continual modification of protein corona due to desorption/adsorption phenomena is called the “Vroman effect” and opens new perspectives for the use of SPIONs in the field of nanomedicine.

In this review, SPIONs neurotoxicity was the focus of numerous studies [75,85,86,88,91,93,114,116,151]. Many authors validated the absence of in vitro toxicity of the SPIONs (if maximal concentration was most often between 200 μg/mL and 500 μg/mL) without cell consequences before inoculating them in animals.

Nevertheless, the risk–benefit balance must be taken into account because absorption, storage and metabolization of SPIONs by different cell types may lead to immediate and chronic immune, inflammatory or metabolic responses [145,152]. Intracellular iron release has potential consequences on cell function and viability. Disturbances of nuclear activity (increased micronuclei and chromosomal condensation), membrane transport blockage, cell membrane rupture (e.g., lactate dehydrogenase leakage through the membrane), induced cell proliferation and abnormal cell growth (genotoxicity), apoptosis (cytotoxicity), and mitochondrial damage (oxidative stress) are among potential toxic effects of SPIONs [152,153,154].

These effects have been observed in rats, 4 days after IV administration of 80 nm SPIONs at 10 mg/kg. The histopathological evaluation showed a diffuse neural degeneration, moderate in hippocampus and striatum, mild in cortex and slight in hypothalamus and pons-medulla areas [155]. In zebrafish, the toxicity of cross-linked aminated dextran (CLIO-NH_2_)-coated SPIONs at different concentrations and exposure times was evaluated through the acetylcholinesterase activity and behavioral observations [88]. At doses above 200 mg/kg for 24 h, acetylcholinesterase activity decreased, and unusual swimming patterns appeared, suggesting a CNS toxicity. These studies underline the importance of determining the maximum load of SPIONs used in vivo to limit the side effects related to intracerebral iron storage.

In vitro SPIONs ototoxicity was studied in EC5V vestibular cell line [44]. SPIONs measuring 100, 200 or 500 nm at concentrations ranging from 7 × 10^6^ to 3 × 10^10^ particles/mL, for 2 days, induced significant apoptosis as judged by flow cytometry analysis. Of note, 500 nm SPIONs appeared less toxic and had a lower intracellular penetration. However, in vivo ototoxicity results were reassuring. Ototoxicity was evaluated by intracochlear administration of SPIONs in guinea pigs and rats and monitoring of the hearing function by auditory brainstem response threshold-shift for up to 30 days [44,45]. Results were encouraging since these studies did not show a hearing loss. This could be related to the quantity of SPIONs administered inside the cochlea, which was not quantified and could be lower than in vitro studies.

Minor or no adverse reactions to SPIONs in many studies encourage further research on their use in drug delivery. However, it is important to remain vigilant since the heterogeneity of materials and protocols hampers comparisons, and the results from in vitro and animal models cannot be directly applied to human.

## 7. Conclusion and Futures Directions

In this review, the contribution of SPIONs for diseases of the brain and of the inner ear are highlighted. Both systems are protected by a barrier (BBB or BPB), which filter the incoming drugs from the blood and have common anatomical characteristics. SPIONs have been engineered to cross these barriers and to deliver drugs to specific structures thanks to molecular probes. The vast range of substances covering or carried by SPIONs allows to increase the biocompatibility and the ability to cross anatomical barriers for specific situations.

One of the major advantages of SPIONs over other NPs is their possible guidance by an EMF which enhances the targeted drug delivery. This increases the local bioavailability of the drug, reduces the total amount of SPIONs required and the potential adverse effects. Despite limited cellular toxicity of SPIONs shown in vitro and in rodents, further studies on the long-term accumulation of SPIONs in the brain and the inner ear need to be conducted.

## Figures and Tables

**Table 1:** Different kind of polymer shell using to coat SPIONs. A: core size with shell (Transmission Electron Microscopy), B: hydrodynamic size with shell (Dynamic Light Scattering), An: naked core size, Bn: naked hydrodynamic size.

Shell Composition	Size (nm)	Experimental Model	Drugs, Agent
Polyethylene-glycol (PEG) [18,40,47,50,56,57,58,59,60,61,62,63,64,65,66,67,68,69,70,71,72]	4–101.6 (A) 8–1000 (B) 7.5–2-10.3 (An) 22 (Bn)	In vitro In vivo (rat, mouse)	Methotrexate, Transferrin, Anti- Transferin receptor ab, Bacterial nanocellulose, anti-IL-1beta-mab, nutlin-3a, Cisplatin, anti-Ape1 siRNA, Temozolomine, anti-nestin ab, Rituximab, Doxorubicin, folic acid, Indocyanine green, O6-benzylguanine, Chlorotoxin-RNAi, Transactivating-transduction protein
Polyethylenimine (PEI) [40,56,57,58,59,73,74]	4–10.3 (A), 11.6–186.5 (B) 10 (An)	In vitro In vivo (rat)	Complementary DNA, Paclitaxel, Bacterial nanocellulose, anti-Ape1 siRNA, Chlorotoxin-RNAi
Poly(lactide-co-glycolide), (PLGA) [43,46,63,74,75,76,77,78,79]	8.4–178.6 (A) 71.8–482.8 (B) 11.5–84.4 (An) 36.8 (Bn)	In vitro In vivo (guinea pig, mouse, rat)	Methotrexate, Paclitaxel, Adipose-derived stem cells, Temozolomide, Doxorubicin, anti-Nestin ab, Transferrin, polysorbate-80, 5-iodo-2-deoxyuridine, Paclitaxel
Polyvinyl alcohols, PVA [80,81,82]	8–12 (A) 30–99.3 (B) 5–10 (An)	In vitro In vivo (mouse)	No
Aniline-co-N-(1-one-butyric acid) aniline (SPAnH) [83]	-	In vitro In vivo (rat)	1,3-bis(2-chloroethyl)-1-nitrosourea
Poly(γ-glutamic acid-co-distearyl γ-glutamate) [76]	106.5 (A) 110 (B)	In vitro In vivo (mouse)	Paclitaxel, Adipose-derived stem cells
Polybutulcyanoacrylate, PBCA [84]	124.5–148.7 (A)	In vitro	Brain-derived neurotrophic factor
Poly-L-lysine [85]	443.4 (B)	In vitro	No
Poly-(dimethylamine-co-epichlorhydrin-co-ethylendiamine), PEA [86]	10 (A) 47.5 (B)	In vitro	No
Chitosan [36,40,59,68,87]	4–6 (A), 40–300 (B), 6–10 (An)	In vitro In vivo (mouse, zebrafish, rat)	anti-Ape1 siRNA, Prednisolone, Chlorotoxin-RNAi, O6-benzylguanine
Dextran, Carboxydextran [80,88,89,90,91,92,93,94,95,96,97,98,99,100,101,102,103,104]	4–130 (A), 23–150 (B) 4.7–10 (An) 144.2–181.2 (Bn)	In vitro In vivo (mouse, zebrafish, rat) Routine clinical use	Quercetin, Transferrin, lipolysaccharide, Folate-Paclitaxel, Rhodamine 123, Epirubicin, cmHsp70.1 mab, recombinant Interleukin-1 receptor antagonist, Doxorubicin, Monocyte-derived-macrophage, Cyclic pentapeptide c -chlorotoxin

**Table 2:** Polymer SPIONs shells derived from amino acids, sugar, and lipids. A: core size with shell (Transmission Electron Microscopy), B: hydrodynamic size with shell (Dynamic Light Scattering), An: naked core size, Bn: naked hydrodynamic size.

Shell Composition	Size (nm)	Experimental Model	Drugs
Amino-Acid			
Glutathione [58,62]	6.8 (A) 11.8–97.8 (B)	In vitro In vivo (rat)	Cisplatin
Glycine [82]	5–10 (An) 75.7–192.1 (B)	In vitro	No
Glutamic Acid [82]	5–10 (An) 75.7–192.1 (B)	In vitro	No
Human Serum Albumin [18,82]	5 (An) 17–192.1 (B)	In vitro In vivo (rat)	Methotrexate
Collagen [82]	5–10 (An) 17–105.8 (B)	In vitro	No
Aminosilane [86]	10 (A) 45.3 (B)	In vitro	No
Spermine [90]	74–110 (B)	In vitro In vivo (mouse)	Transferrin
**Sugar**			
D-mannose [85]	101.1 (B)	In vitro	No
Hydrophilic polysaccharide matrix of starch (α-D-glucose units) [111]	117.4 (B)	In vitro In vivo (rat)	No
Rhamnose [109]	19.4 (A)	In vitro	No
nGum arabic [110]	14 (A) 100 (B)	In vitro In vivo (rat)	Rhodamine B
**Lipid**			
Oleic acid [76,82,112]	5.2–106.5 (A) 110 (B)	In vitro In vivo (mouse)	Paclitaxel, Adipose-derived and mesenchymal stem cells, Rhodamine B
Micelles [48,62]	5–6.8 (A) 7–100 (B)	In vitro In vivo (rat)	Cisplatin, Lactoferrin
1,2-Distearoyl-sn-glycero-3-phosphoethanolamine, DSPE [50,113]	36–80 (A) 20 (An) 94.7 (B)	In vitro In vivo (mouse)	Epigallocatechin gallate, Temozolomine, anti-transferin receptor ab
Phospholipid [66]	9.8–22.9 (B)	In vitro In vivo (rat, mouse)	Doxorubicin, Indocyanine green
Dimyristoyl-phophatidyl-choline, DMPC [69]	31 (B)	In vitro In vivo (rat)	No
Lipophilic fluorescence dye [111]	117.4 (B)	In vitro In vivo (rat)	No

**Table 3:** Miscellaneous SPIONs shells A: core size with shell (Transmission Electron Microscopy), B: hydrodynamic size with shell (Dynamic Light Scattering), An: naked core size, Bn: naked hydrodynamic size.

Shell Composition	Size (nm)	Experimental Model	Drugs
Molecular Ligands			
Curcumin [48,116,117]	5–122.2 (A) 7–185 (B) 11 (An)	In vitro In vivo (rat, mousse)	Lactoferrin, RGE peptide (a specific ligand of NPR-1)
dimercaptosuccinic acid-DMSA [118]	4–9 (A) 65–70 (B)	In vitro In vivo (rat)	No
Liposome [47,64,71,101,119,120]	7–104 (A) 83.2–190 (B) 5–10 (An) 7.4 (Bn)	In vitro In vivo (rat)	Paclitaxel, Transactivating-transduction protein, Doxorubicin, Rituximab, Transferrin
**Surfactants**			
Janus [65]	90.4 (A)	In vitro	Doxorubicin, folic acid
Amphiphilic polymer [102]	10–30 (A) 18–40 (B)	In vitro In vivo (rat)	Monocyte-derived-macrophage
TPGS [75]	178.6 (A) 29.9 (An)	In vitro In vivo (mouse)	No
D-Alpha-Tocopheryl Polyethylene Glycol Succinate [77]	8.4 (A) 227.9 (B)	In vitro	Doxorubicin
DMAB [75]	67.1 (A) 29.9 (An)	In vitro In vivo (mouse)	No
Twen 80 [46,49,57,64,77,84]	8.4–148.7 (A) 140–220 (B)	In vitro In vivo (rat, mouse)	Clonazepam, Brain-derived neurotrophic factor, Temozolomide, Doxorubicin, Rituximab

## Data Availability

Not applicable.
